# Chemical Imaging on Liver Steatosis Using Synchrotron Infrared and ToF-SIMS Microspectroscopies

**DOI:** 10.1371/journal.pone.0007408

**Published:** 2009-10-12

**Authors:** François Le Naour, Marie-Pierre Bralet, Delphine Debois, Christophe Sandt, Catherine Guettier, Paul Dumas, Alain Brunelle, Olivier Laprévote

**Affiliations:** 1 Inserm U602, Villejuif, France; 2 Université Paris-Sud, Institut André Lwoff, Villejuif, France; 3 Inserm U785, Villejuif, France; 4 Service d'anatomie pathologique, Hôpital Paul Brousse, Villejuif, France; 5 Institut de Chimie des Substances Naturelles, CNRS, UPR 2301, Gif-sur-Yvette, France; 6 Synchrotron SOLEIL, Gif-sur-Yvette, France; 7 Laboratoire de Toxicologie, IFR 71, Faculté des Sciences Pharmaceutiques et Biologiques, Université Paris-Descartes, Paris, France; HelmholtzZentrum München, Germany

## Abstract

Fatty liver or steatosis is a frequent histopathological change. It is a precursor for steatohepatitis that may progress to cirrhosis and in some cases to hepatocellular carcinoma. In this study we addressed the *in situ* composition and distribution of biochemical compounds on tissue sections of steatotic liver using both synchrotron FTIR (Fourier transform infrared) and ToF-SIMS (time of flight secondary ion mass spectrometry) microspectroscopies. FTIR is a vibrational spectroscopy that allows investigating the global biochemical composition and ToF-SIMS lead to identify molecular species in particular lipids. Synchrotron FTIR microspectroscopy demonstrated that bands linked to lipid contribution such as -CH_3_ and -CH_2_ as well as esters were highly intense in steatotic vesicles. Moreover, a careful analysis of the -CH_2_ symmetric and anti-symmetric stretching modes revealed a slight downward shift in spectra recorded inside steatotic vesicles when compared to spectra recorded outside, suggesting a different lipid environment inside the steatotic vesicles. ToF-SIMS analysis of such steatotic vesicles disclosed a selective enrichment in cholesterol as well as in diacylglycerol (DAG) species carrying long alkyl chains. Indeed, DAG C36 species were selectively localized inside the steatotic vesicles whereas DAG C30 species were detected mostly outside. Furthermore, FTIR detected a signal corresponding to olefin (C = C, 3000-3060 cm^−1^) and revealed a selective localization of unsaturated lipids inside the steatotic vesicles. ToF-SIMS analysis definitely demonstrated that DAG species C30, C32, C34 and C36 carrying at least one unsaturated alkyl chain were selectively concentrated into the steatotic vesicles. On the other hand, investigations performed on the non-steatotic part of the fatty livers have revealed important changes when compared to the normal liver. Although the non-steatotic regions of fatty livers exhibited normal histological aspect, IR spectra demonstrated an increase in the lipid content and ToF-SIMS detected small lipid droplets corresponding most likely to the first steps of lipid accretion.

## Introduction

Fatty liver or steatosis is a frequent histopathological change resulting from a wide spectrum of clinical conditions such as alcoholism, drug intake, small-bowel by-pass surgery or metabolic syndrome. Non alcoholic fatty liver disease known to be associated with obesity, insulin resistance, diabetes, drugs and the metabolic syndrome is probably the most common cause of chronic liver disease in Western countries. It is now clear that fatty liver is a precursor for steatohepatitis, a condition that may progress to cirrhosis and in some cases to the development of primary liver cancer [1]. The hallmark feature of fatty liver disease is the intra-cellular accumulation of triacylglycerol (TAG) and diacylglycerol (DAG) resulting in the formation of steatotic vesicles in the hepatocytes. This accumulation results from an imbalance in the uptake, synthesis, export and oxidation of fatty acids [2], [3]. However, the primary metabolic abnormalities leading to lipid accretion are not well understood and the local lipid composition has been poorly studied.

Imaging techniques based on spectroscopy such as infrared spectroscopy or mass spectrometry have been developed or improved since the last ten years. Infrared spectroscopy is based on the determination of absorption of infrared light due to resonance with vibrational motions of functional molecular groups. Biological tissue is essentially made up of proteins, nucleic acids, carbohydrates and lipids all of which have characteristics absorption bands in the infrared frequency domain. As such infrared spectroscopy is a very valuable tool for biochemical investigations. Fourier Transform Infrared (FTIR) microspectroscopy combines IR spectroscopy and microscopy for determining the chemical composition in small sample area. Application of synchrotron radiation as a high brightness source of infrared photons has brought the technique to achieve analysis at the diffraction limit (typically, half the wavelength of the vibrational frequency) while preserving a high spectral quality [4], [5]. On the other hand, imaging techniques based on mass spectrometry allow the mapping of compounds present at the surface of a tissue section. Time-of-Flight-Secondary Ion Mass Spectrometry (ToF-SIMS) uses a pulsed and focused primary ion beam (often clusters of heavy metals) to desorb and ionize species from the sample surface. The resulting secondary ions are extracted towards a mass spectrometer, where they are mass analyzed by measuring their time-of-flight (ToF) from the sample surface to the detector. Ions are identified according to their mass-to-charge (*m/z*) ratio. The accessible mass range is about 1500 Da, which makes this technique very suitable for small molecule analysis such as lipids. Chemical images are generated by collecting a mass spectrum at every pixel as a finely focused primary ion beam is rastered across the sample surface. The mass spectrum and the secondary ion images are then used to determine the composition and distribution of sample surface constituents [6]–[8]. A major advantage of FTIR and ToF-SIMS imaging techniques is that they do not necessitate any matrix deposition, chemical treatment or staining to acquire images. In addition, the spatial resolution of both imaging techniques allows working at cellular and sub-cellular levels. Thus, for the last years FTIR as well as ToF-SIMS were used in various biological and clinical investigations [4]–[9]. However, very few studies have been performed with the combination of both techniques.

Dramatic changes in terms of biochemical composition characterize liver steatosis. Investigating these changes may provide new insights into the understanding of mechanisms underlying fatty liver diseases and into identifying markers for diagnosis and prognosis. In this study, FTIR microspectroscopy using synchrotron radiation and ToF-SIMS have been employed to perform chemical imaging of steatosis. This has allowed visualization of important variations in lipid composition and environment into steatotic hepatocytes. Furthermore, both spectroscopies revealed important changes of the non-steatotic part of fatty livers despite normal histological aspect.

## Materials and Methods

### Ethics statement

The study was approved by INSERM and Hôpital Paul Brousse. The patients were informed and their consent was obtained and written. The ethics committee specifically approved that procedure.

### Patients and liver samples

Liver specimens were obtained from the Centre de Ressources Biologiques Paris-Sud, Paris-Sud 11 University, France. Access to this material was in agreement with French laws. Tissue samples were obtained from the non-tumoral part of six liver resection specimens (Table 1). For all patients, daily alcohol consumption was lower than 30 g for men and 20 g for women. Infection with hepatitis B virus (HBV) or hepatitis C virus (HCV); genetic hemochromatosis; autoimmune liver diseases, Wilson's disease were excluded. Tissues were fixed in formalin for routine pathological assessment and one specimen of non-tumorous liver distant to tumor was immediately snap frozen in liquid nitrogen and stored at −80°C until use. For three patients, liver was histologically normal. For three other patients, microscopic analysis revealed bland macrovesicular and microvesicular steatosis without hepatocyte ballooning, lobular inflammation, perisinusoidal fibrosis, nor Mallory's hyaline.

**Table 1:** History of patients and origin of samples.

Patient	Sex	Age	Pathological diagnosis	Associated diagnosis	Macrovacuolar steatosis (%)	Microvesicular steatosis (%)
1	F	32	Normal liver	Focal nodular hyperplasia	0	0
2	F	25	Normal liver	Focal nodular hyperplasia	0	0
3	F	41	Normal liver	Focal nodular hyperplasia	0	0
4	F	57	Steatosis	Gallbladder carcinoma	20	10
5	M	58	Steatosis	Liver metastasis from colorectal cancer	20	10
6	F	65	Steatosis	Liver metastasis from breast cancer	20	5

### Tissue section

**Figure 1:**
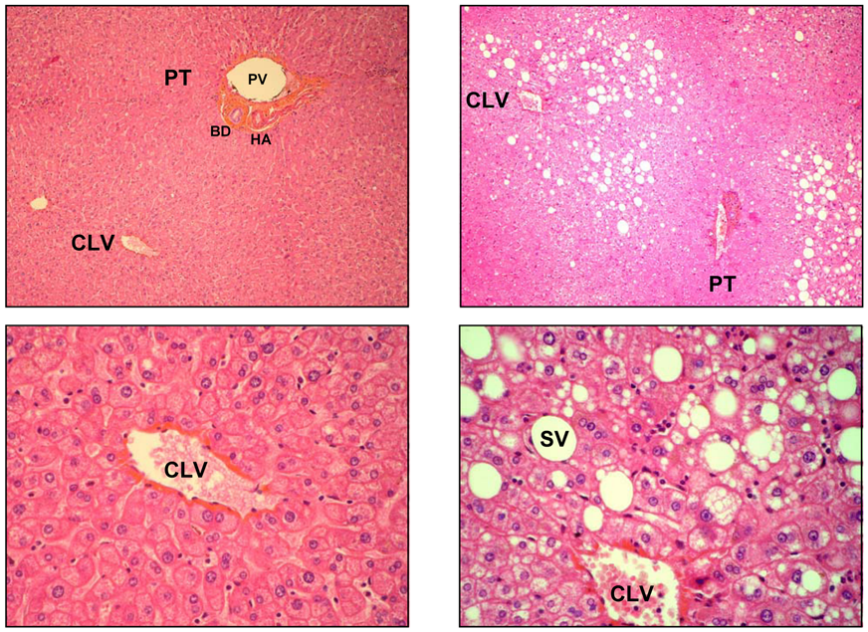
Histological features of steatosis. Tissue sections of 6 µm thickness were performed on paraffin embedded biopsies from normal liver or from fatty liver and stained with HES (hematoxylin, eosin and safran). Normal hepatic lobule without steatosis (left panel) or fatty liver area exhibiting macrovacuolar and microvesicular steatosis (right panel) are shown. Upper panel: ×100, lower panel: ×400. PT: portal tract, BD: biliary duct, PV: portal vein, HA: hepatic artery, CLV: centrilobular vein, SV: steatotic vacuole.

Serial sections were cut with 6–10 µm thick at −20°C with a CM3050-S cryostat (Leica Microsystèmes SAS, France) and alternately deposited on glass slide for extemporaneous histological control and on glass slide (Tientascience, Indianapolis, IN) for FTIR microspectroscopy or on silicon wafer (2 inch diameter polished silicon wafers, ACM, Villiers-Saint-Frédéric, France) for ToF-SIMS imaging. Sections for histology were stained with hematoxylin eosin saffron (HES) (Fig. 1). Sections for FTIR microspectroscopy were dried a few min at room temperature and those for ToF-SIMS imaging were dried under a pressure of a few hPa for 30 min.

### Synchrotron FTIR microspectroscopy

Synchrotron infrared micro-spectroscopy was performed at the SMIS beamline at the SOLEIL synchrotron facility (Saint Aubin, France). The beamline is exploiting the bending magnet radiation of the synchrotron radiation, which is coupled to a Thermo Fischer NEXUS FTIR spectrometer Nicolet 5700. Attached to the spectrometer is a microscope CONTINUUM XL (Thermo Scientist, CA). The detector of the infrared microscope is a liquid nitrogen cooled mercury cadmium telluride (MCT-A) detector (50 µm). The microscope was operating in confocal mode, using a 32× infinity corrected Schwarzschild objective (NA = 0.65) and a matching 32× condenser. All spectra were obtained using a double path single masking aperture (confocal arrangement) size ranging from 6×6 µm^2^ to 12×12 µm^2^. The brightness advantage of the synchrotron infrared source with this configuration at the SMIS beamline was about 60 with a 10×10 µm^2^ aperture and 110 with a 6×6 µm^2^ aperture compared to globar source. The signal to noise ratio was 0.04% with the synchrotron source whereas it was 2% at 10×10 µm^2^ aperture with a globar source. The spectra were collected in the 4000–800 cm^−1^ mid-infrared range at a resolution of 4 cm^−1^ with 50 co-added scans. Data analysis of IR spectra and chemical images were performed using OMNIC software (Thermo Scientific).

### ToF-SIMS Imaging

A standard commercial ToF-SIMS IV (Ion-Tof GmbH, Münster, Germany) reflectron-type TOF mass spectrometer was used for mass spectrometry imaging experiments. The analysis was performed as previously described [10], [11]. Briefly, the primary ion source was a bismuth liquid metal ion gun. Bi_3_^+^ cluster ions were selected. The ion column focusing mode ensured a spatial resolution of 1–2 µm and a mass resolution M/ΔM = 10^4^ (full width half maximum, FWHM) at *m/z* 500. The mass calibration was always internal and signals used for initial calibration were those of H^+^, H_2_^+^, H_3_^+^, C^+^, CH^+^, CH_2_^+^, CH_3_^+^ for the positive ion mode. Signals from compounds such as cholesterol and diacylglycerols were used for calibration refinement. Structure attributions or assignments of ion peaks were made according to the instrument resolution, accuracy and the valence rule and the biological relevance of the attribution (according to the tissue type for instance). Ion images were recorded for each selected area with a primary ion fluence of 3.10^11^ ions.cm^−2^. Images were recorded with a field of view of 500×500 µm^2^ and 256×256 pixels, giving a pixel size of 2×2 µm^2^. Image reconstruction was done by integrating signal intensities at desired *m/z* values across the data set. A color scale bar, for which the amplitude, in counts, is given for each image, is placed to the right of the ion images. The data acquisition and processing softwares were IonSpec and IonImage (Ion-Tof GmbH, Münster, Germany). Regions Of Interest (ROIs) were manually selected with the imaging software. The associated mass spectra were further extracted in order to obtain the subsequent local information, leading to more precise localizations and relative intensities. For a proper and easier comparison, as each ROI had a different area (in pixels), a normalization of their respective mass spectrum intensities had to be performed. The intensity of the mass spectrum from each ROI was normalized as if it was composed of the same number of pixels as the smallest one.

## Results

### Chemical imaging on steatosis using FTIR microspectroscopy

**Figure 2:**
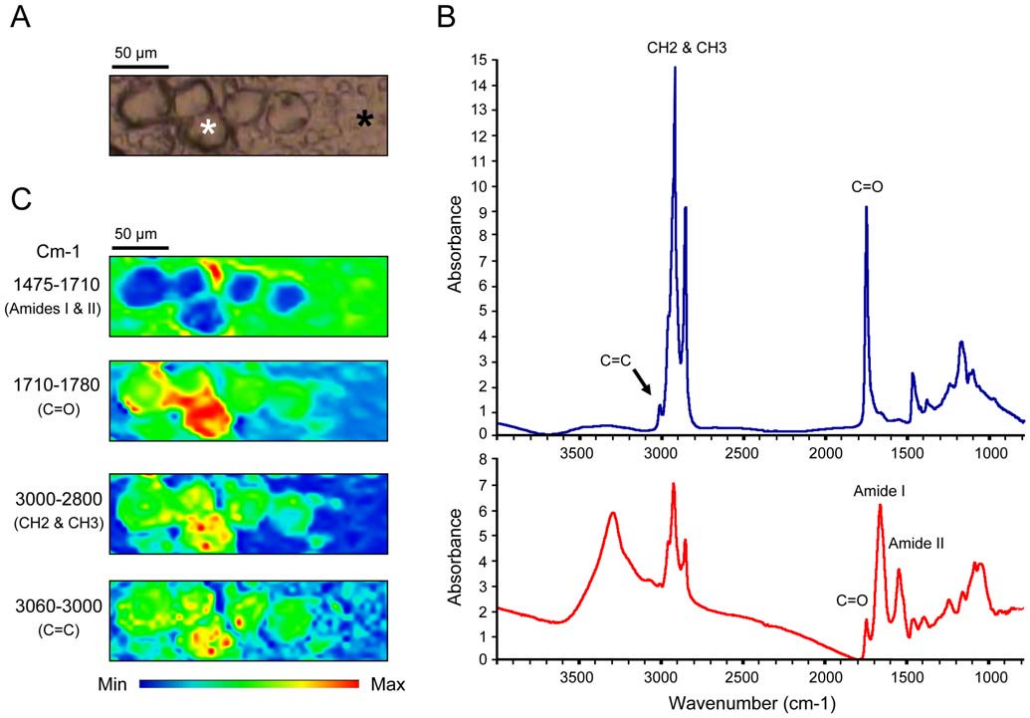
Analysis of steatosis using synchrotron FTIR microspectroscopy. A) Optical image of steatotic hepatocytes containing steatotic vesicles (white star) and non-steatotic hepatocytes (black star). B) Averaged IR spectra recorded inside steatotic vesicles (upper spectrum in blue) or on non-steatotic hepatocytes (lower spectrum in red). The band corresponding to olefin (3000–3060 cm^−1^) is labelled by a black arrow. C) Chemical imaging of some bands on the tissue section.

**Figure 3:**
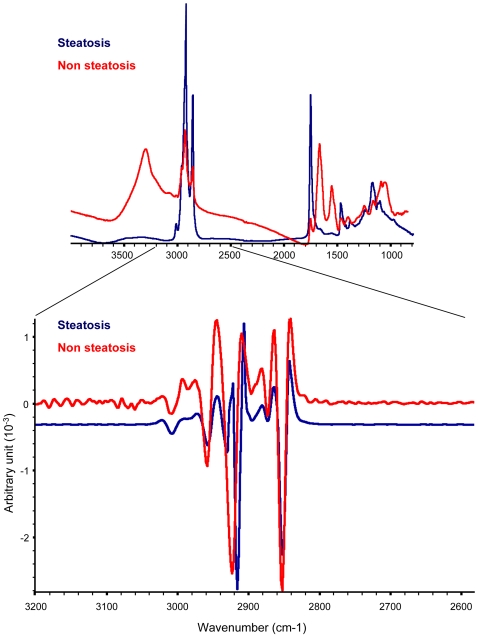
Second derivatives of IR spectra. Spectra recorded on steatosis or non-steatotic hepatocytes were superimposed (upper panel). Second derivatives of the spectra were calculated and superimposed in the frequency domain 2600–3200 cm^−1^ (lower panel).

Nine different regions were selected from 3 steatotic livers exhibiting macrovacuolar and microvesicular steatosis (Table 1, Fig. 1). Steatosis and non-steaotic regions of fatty livers were investigated by FTIR microspectroscopy. We employed a synchrotron infrared source that provides in the mid-IR domain a bright source. The brightness has lead to improve the lateral resolution (less than 10×10 µm^2^) while conserving good signal to noise ratio. Thus, the bright synchrotron infrared source allows recording several spectra inside a single steatotic vesicle (30 µm diameter). The spectra exhibited marked changes compared to those recorded in non-steatotic hepatocytes. In order to characterize the main differences between these two regions, more spectra were acquired in steatotic vesicles or non-steatotic regions. In each region, spectra have been found very similar. They were further averaged (Fig. 2A, B). The spectral bands that can be assigned to chemical functions or to the contribution of macromolecules are reported in Table 2. The comparison of the two averaged spectra obtained on steatotic and non-steatotic hepatocytes allows observing that proteins, characterized by Amide I and II bands centred respectively at 1650 and 1540 cm^−1^, were not detected in the steatotic vesicles. As expected, major changes were observed in the lipid frequency domains, such as the relative intensity of the -CH_3_ and -CH_2_ (3000–2800 cm^−1^) and of the ester signals (C = O, 1740 cm^−1^) which increased significantly in steatosis. Interestingly, a band corresponding to olefin (C = C, 3060–3000 cm^−1^) was detected only in steatosis. This peak corresponds to unsaturated carbon chains (Fig. 2B). Furthermore, the relative distributions of the main biochemical compounds such as proteins and lipids were investigated by raster scanning the sample with 5 micron steps, and recording infrared spectra at each pixel (10×10 µm^2^). The total absorbance of each characteristic band has been calculated to reconstruct a chemical image of the sample probed. The chemical image of the proteins, generated in the frequency region 1475–1710 cm^−1^ (Amide I and II bands) showed the proteins surrounding the steatotic vesicles, but not present inside. By contrast, the vesicles contained a much higher concentration of lipids, as observed by integrating the frequency region of the stretching motions of -CH_2_ and -CH_3_ (2800–3000 cm^−1^). Interestingly, the chemical image of unsaturated lipids, in the frequency region 3000–3060 cm^−1^, clearly demonstrated a selective localization inside the steatotic vesicles as well as the distribution of ester bands, generated in the frequency region 1710–1760 cm^−1^ (Fig. 2C). Moreover, an interesting observation raised from a careful analysis of the CH_2_ symmetric and anti-symmetric stretching modes, recorded inside the steatotic vesicles and outside (Fig. 3). Indeed, a slight downward shift of the CH_2_ symmetric and anti-symmetric stretching modes was observed in spectra recorded in steatosis vesicles. This has been more clearly determined by displaying the second derivative of the raw spectra as shown on Fig 3B. This downward shift has an origin in the local organization of lipids [9]. Thus, the lipids inside the steatotic vesicles are in a different environment, probably with a higher structural order.

**Table 2:** Assignment of frequency to chemical functions.

Frequency (cm^−1^)	Chemical function
∼3500	O-H stretch of hydroxyl groups
∼3200	N-H stretch ( Amide A) of proteins
∼3000–3060	C = C stretch
∼2955	C-H asymmetric stretch of –CH_3_ in fatty acids
∼2930	C-H asymmetric stretch of –CH2
∼2918	C-H asymmetric stretch in fatty acids
∼2898	C-H symmetric stretch of C-H in methyl groups
∼2870	C-H symmetric stretch of –CH3
∼2850	C-H symmetric stretch of –CH2 in fatty acids
∼1740	-C = O stretch of esters
∼1715	-C = O stretch of carbonic acids
∼1680–1710	-C = O in nucleic acids
∼1695	Amide I band components of proteins
∼1685	Antiparallel pleated sheets
∼1675	Resulting from β- turns of proteins
∼1655	Amide I of α-helical structures
∼1635	Amide I from β-pleated sheet structures
∼1575	Asymmetric stretch of COO-
∼1550–1520	Amide II
∼1515	“Tyrosine” band
∼1468	C-H deformation of –CH_2_
∼1400	C = O symmetric stretch of COO-
∼1310–1240	Amide III band components of proteins
∼1250–1220	P = O asymmetric stretch of PO_2_^−^ phosphodiesters
∼1200–900	C-O; C-C; C-O-H; C-O-C deformation of carbohydrates
∼1090–1085	P = O symmetric stretch of PO_2_^−^
∼720	C-H rocking of –CH_2_
∼900–600	“ Fingerprint region”

From [19], [20].

### Chemical imaging on steatosis using ToF-SIMS mass spectrometry

**Figure 4:**
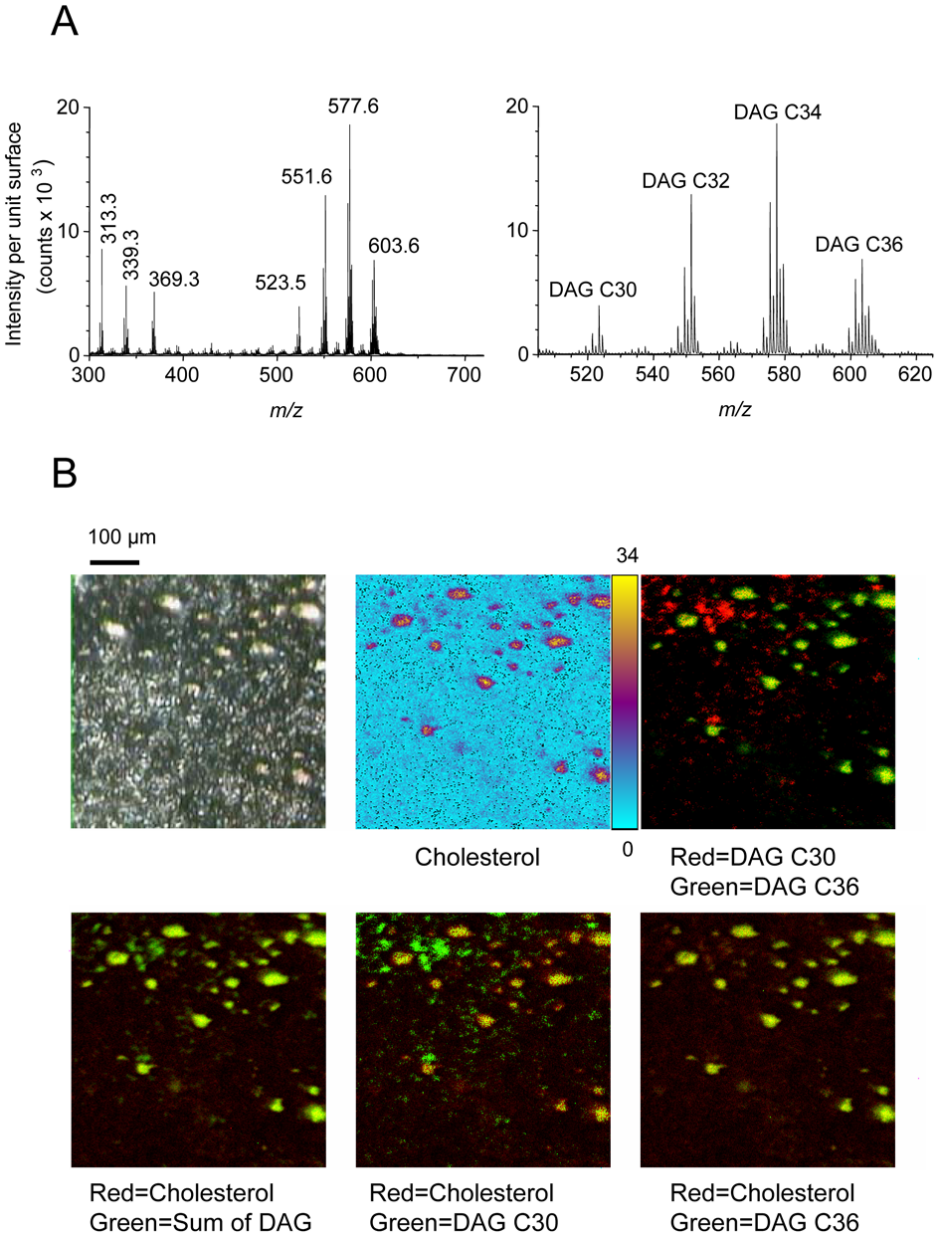
Mass spectrometry chemical imaging on steatosis using ToF-SIMS. A) ToF-SIMS mass spectra were extracted from steatotic regions on fatty liver. Positive ion mode allowed detecting monoacylglycerol species (*m/z* 313.3 and *m/z* 339.3), cholesterol (*m/z* 369.3), diacylglycerol species C30 (*m/z* 523.5), C32 (*m/z* 551.6), C34 (*m/z* 577.6) and C36 (*m/z* 603.6). B) ToF-SIMS imaging of a steatotic region was performed. The video image is shown (upper left) as well as the distribution of cholesterol (upper middle). The maximum ion count recorded on a pixel in the image is indicated on the color scale bar. The selective distribution of DAG species C30 and C36 was confirmed by two color overlays (upper right). The selective distribution of cholesterol and DAG species was investigated by two color overlays (lower panel).

**Figure 5:**
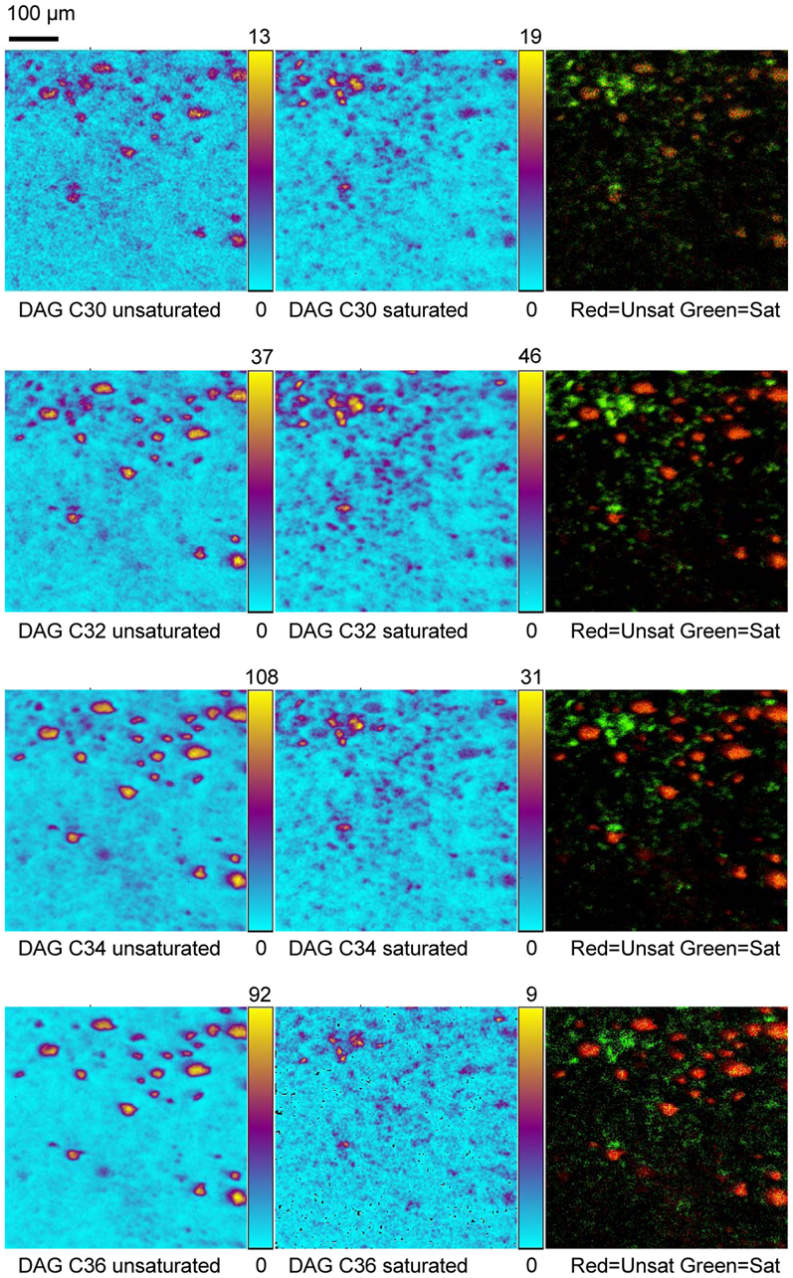
Mass spectrometry imaging of DAG species using ToF-SIMS. The selective distribution of DAG species carrying unsaturated or saturated alkyl chains were investigated and confirmed by two color overlays.

In order to investigate the local variation of the molecular composition and environment in steatosis, mass spectrometry experiments were performed using ToF-SIMS (time-of-flight secondary ion mass spectrometry). This spectroscopic approach is suitable for the characterization of lipid composition on tissue section without any matrix deposition or chemical treatment. In addition, ToF-SIMS allows imaging lipid species for determining their localization at cellular and sub-cellular levels. Thus, additional tissue sections analyses were performed from a steatotic liver previously analyzed using FTIR microspectroscopy. Steatotic regions were selected and mass spectra were acquired using ToF-SIMS mass spectrometry in the positive ionization mode (Fig. 4A). Several lipids were detected as highly abundant species such as cholesterol, monoacylglycerols (MAG) and diacylglycerols (DAG). It should be noted that the presence of MAG may result from the fragmentation of DAG and TAG since ToF-SIMS is known to induce such molecular fragmentations [8], [10], [11]. The distribution of some detected lipids was addressed precisely by imaging mass spectrometry. Cholesterol exhibited selective macrovacuolar distribution (Fig. 4B). Localizations of the various DAG species were investigated revealing important differences in their distribution. Thus, DAG C30 species were detected mostly outside of steatotic vesicles whereas DAG C36 species were selectively localized in these vesicles. Overlay of respective ion images allowed distinguishing different localization of these two molecular species since they appear in two different colors (red and green). Furthermore, overlay of ion images of DAG C36 species and cholesterol definitely demonstrated the co-localization of these lipids in steatotic vesicles which appeared in yellow (Fig. 4B). The distribution of DAG species carrying saturated or unsaturated alkyl chains were also investigated. DAG C30, C32, C34 and C36 bearing saturated alkyl chains were selectively located outside of the steatotic vesicles whereas these DAG species containing at least one unsaturated acyl chain were selectively concentrated into steatotic droplets. These anti-correlated locations were confirmed by overlay images (Fig. 5).

### Spectroscopic analysis of non-steatotic hepatocytes in fatty liver

**Figure 6:**
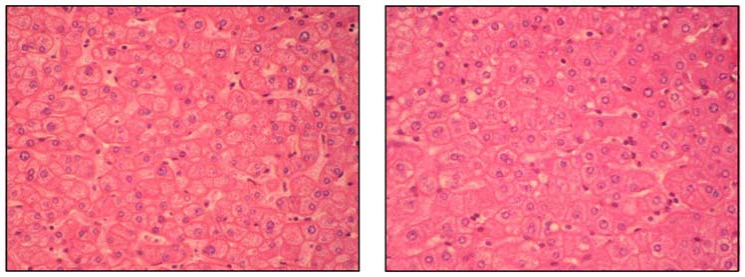
Histological aspects of normal liver and non-steatotic area from fatty liver. Tissue sections of 6 µm thickness were performed on paraffin embedded biopsies from normal liver (right) or from non-steatotic area of fatty liver (left) and stained with HES (hematoxylin, eosin and safran) (x400).

**Figure 7:**
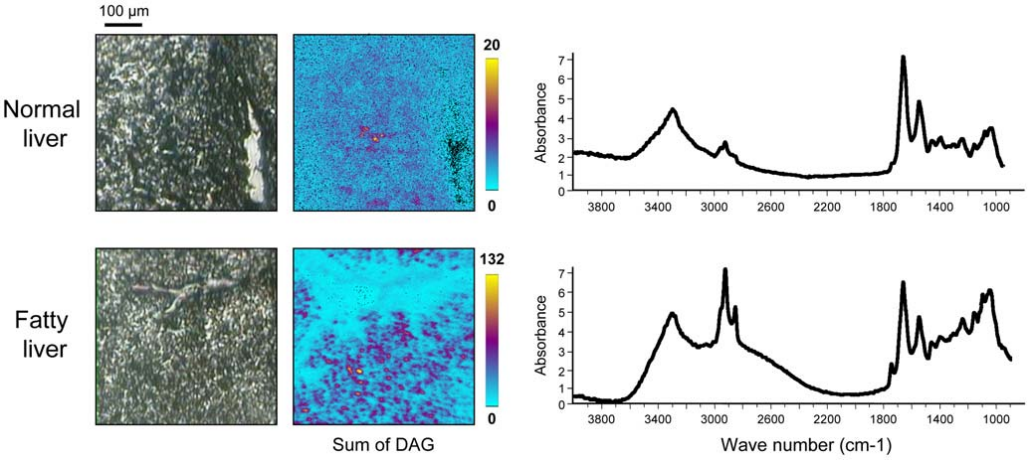
Spectroscopic analysis of non-steatotic hepatocytes on fatty liver. Spectroscopic analyses were performed on periportal hepatocytes on tissue section from normal or fatty liver. The video image is shown (left panel) with the corresponding averaged IR spectra (right panel) and the chemical imaging of the sum of DAG (middle panel).

In order to compare the non-steatotic part of fatty liver to the normal liver, investigations have been performed using both synchrotron FTIR and ToF-SIMS microspectroscopies. Biopsies from three fatty livers and three normal livers were used in these experiments (Table 1). Serial tissue sections were obtained on both fatty and normal livers. Examination of the non-steatotic areas of fatty livers did not exhibit major histological changes when compared to the normal livers (Fig. 6). By contrast, synchrotron FTIR spectroscopy revealed important changes in the IR spectra acquired on non-steatotic regions of fatty liver and normal liver. The major changes were observed on the lipid content as demonstrated by the higher intensity of CH_3_ and CH_2_ bands (2800–3000 cm^−1^) and esters (1710–1760 cm^−1^) in IR spectra recorded from non-steatotic hepatocytes of fatty liver. Interestingly, changes were also observed on bands in the frequency domains 950–1200 cm^−1^ corresponding in part to sugar contribution. ToF-SIMS analyses were performed on some serial tissue sections. These analyses on non-steatotic hepatocytes of fatty liver demonstrated the increase in the lipid content and allowed visualizing the presence of small lipid droplets exhibiting sizes less than 10 µm for most of them, containing DAGs. These droplets correspond most likely to the first events of lipid accretion (Fig. 7). Therefore, these observations demonstrated that hepatocytes looking like non-steatotic in fatty liver exhibit metabolic disturbance and are qualitatively different to normal hepatocytes. Spectroscopic approaches are then suitable to reveal such metabolic disturbance at early stages.

## Discussion

In this study, we addressed the in situ composition and distribution of biochemical compounds on tissue sections from biopsies of steatotic liver using two types of microspectroscopies: synchrotron infrared and ToF-SIMS microspectroscopies. Few infrared microspectroscopy studies have been already carried out on liver sections [12]–[14], and they concentrated mainly on the frequency region between 900 and 1800 cm^−1^, using an internal source. In our study, we employed for the first time the synchrotron source for such a study, in order to increase markedly the spatial resolution, and to study the complete frequency range from 900 to 4000 cm^−1^, where the fingerprints of the lipids lie. In addition, we have employed ToF-SIMS for investigating the local composition and distribution of the molecular species of lipids. Both techniques can be performed directly on tissue section and do not necessitate any treatment. Moreover, they exhibit similar high spatial resolution allowing investigation at cellular and subcellular levels. Infrared microspectroscopy leads to address the global composition of the tissue whereas ToF-SIMS allows investigation of lipid profile. A major interest of combining infrared spectroscopy with mass spectrometry is the possibility to establish a link between IR spectra and the molecular composition.

With regards to steatosis, synchrotron FTIR microspectroscopy revealed the appearance of unsaturated lipids inside steatotic vesicles in a probably higher structural ordered lipid environment. ToF-SIMS allowed analyzing the composition of such steatotic vesicles thus demonstrating a selective enrichment in cholesterol and DAG species carrying unsaturated alkyl chains. Thus, both spectroscopies demonstrated that dramatic changes of lipid composition occur during steatosis in addition to lipid accumulation. These findings raise the question of the formation of the lipid droplets during the course of steatosis. Indeed, the selective concentration of cholesterol with DAG species carrying long and unsaturated alkyl chains may result of the passive accretion of these lipids based on their physicochemical properties. Inversely, this phenomenon may result of an active process involving energetic metabolism and enzymes. The study of the molecular mechanisms underlying the formation of lipid droplets may give new insights in the understanding of steatosis and will have to be addressed in further studies. On the other hand, the concentration of unsaturated lipids inside steatotic vesicles may constitute a potential highly reactive site for peroxidation. Given that lipid peroxidation is based on a radical reaction that is propagating by chain reaction, the concentration of reactive molecules may dramatically increase the impact of the peroxidation reaction and the resulting molecular and cellular damages in the liver [15]–[17].

Finally, investigations performed on the non-steatotic areas of the fatty livers using both synchrotron FTIR and ToF-SIMS microspectroscopies have revealed important changes when compared to the normal liver. Although the non-steatotic regions were identical to normal liver on standard microscopy analysis, an increase in the lipid content leading to the formation of small lipid droplets was detected in the hepatocytes. The presence of these small lipid droplets may correspond to early metabolic disturbance preceding steatosis or to microvesicular steatosis undetected by standard microscopy. As microvesicular steatosis is not a benign condition and is associated with reduced regenerative capacity of the liver [18], infrared spectroscopy might be used as a diagnosis mean especially in the setting of liver transplantation, thus allowing a rapid statement on the quality of a potential liver graft.

In conclusion, this study emphasizes the advantages of combining different spectroscopies for investigating *in situ* the chemical composition of tissues. The spatial resolution and sensitivity of synchrotron FTIR microspectroscopy and mass spectrometry may open new avenue for characterizing early events occurring in pathologies or for identifying markers for diagnosis and prognosis. Once such markers identified, FTIR microspectroscopy using conventional infrared source might be set up in hospitals for clinical use.
